# An On-Chip Bandpass Filter Using Complementary Slit-Ring-Resonator-Loaded Spoof Surface Plasmon Polaritons with a Flexible Notch-Band

**DOI:** 10.3390/mi14030607

**Published:** 2023-03-06

**Authors:** Xin Cao, Chenyi Wang, Weiping Li, Qiangming Cai

**Affiliations:** 1 School of Information Engineering, Southwest University of Science and Technology, Mianyang 621010, China; 2 School of Information Engineering, East China Jiaotong University, Nanchang 330013, China

**Keywords:** spoof surface plasmon polariton, complementary slit-ring resonator, bandpass filter, notch-band, 5G communication

## Abstract

In this paper, an on-chip bandpass filter with enhanced notch-band is proposed based on the artificial spoof surface plasmon polariton (SSPP) structure around 60 GHz. The shapes of the tabs are modified to incorporate complementary slit-ring resonators (CSRRs) between tabs. The passband is produced by the first higher mode of the SSPP structure. By adding CSRRs, two notch frequencies have been produced in the passband. The advantage of the proposed structure is that these two transmission zeros are almost independent of each other and they do not have much impact on the frequency of the passband. Based on the measured results, the proposed filter has an insertion loss of 2.6 dB with the bandwidth of 14.3 GHz. The return loss is better than 12 dB. The notch-band has a bandwidth of 3.3 GHz with a suppression level over 20 dB. The proposed filter can be applied in the shared millimeter waveband of 5G communication systems.

## 1. Introduction

The development of 5G communication has opened a new era of mobile communication systems. It is not enough to rely only on the evolution of low-frequency (Sub-6G) wireless transmission and networking technology. The communication frequency band must be extended to the millimeter-wave frequency band. The number of filters that interfere with each other between isolated frequency bands in the link will be greatly increased. The development of filters is moving towards the characteristics of miniaturization, higher integration level, and higher operating frequency. Especially when developing to the millimeter-wave frequency band, traditional planar transmission lines, such as microstrip lines, are not suitable because of their high transmission loss and coupling effect. Surface plasmon polaritons (SPPs) were proposed in [1,2,3,4,5,6]. This structure has some special properties, such as strong dispersion, strong field confinement, high frequency cutoff, and local field enhancement. It can not only further compress the waveguide wavelength to achieve miniaturization, but also avoid interference between transmission lines. Furthermore, it is especially suitable for high-frequency application scenarios. SSPPs have become a very hot research area in recent years.

The most common way to realize a bandpass filter using the SSPP structure is to couple the SSPP to the feed lines directly. SSPPs can be directly coupled to transmission lines with each other, which can produce a passband effect [7,8,9,10]. The passband range is determined by the length of the coupling segment of the SSPPs’ transmission line. Due to the strong field binding and cut-off characteristics of SSPPs, the parasitic passband on the right transition bands can be suppressed. Since this structure is relatively simple, the parasitic passbands on the left transition band are difficult to suppress. In addition, this kind of coupling structure usually results in higher insertion loss.

To solve this problem, some researchers have added some gap or branch structures at both ends of the SSPPs’ transmission line to increase low-frequency suppression. A resonant ring can be added in the middle of the SSPPs’ transmission line [11,12,13,14] so that the electromagnetic energy can be absorbed at the center frequency of the left parasitic passband. Another type of structure that combines substrate integrated waveguide (SIW) with SSPPs, known as a hybrid SIW-SSPP structure, has also been proposed [15,16,17]. By employing the low-pass characteristic of SSPPs and the high-pass characteristic of SIWs, the upper and lower frequencies of the transition bands have been improved at the same time. However, in real applications, impedance-matching networks should be added at the input and output of the SIW structure, which can cause additional loss.

Another method is to integrate with the substrate combined with the high-pass properties of the waveguide. A series of studies on etching SSPPs into substrate integrated waveguides have been proposed. The SSPPs combined with the half-mode substrate integrated waveguide have been proposed in [18,19]. It can reduce the size to half of the original size. This design method has very excellent out-of-band rejection and roll-off coefficient, and the insertion loss is relatively small. However, the edge of the passband is prone to jitter and is not smooth enough. Furthermore, by loading some metal resonant cavities or gaps at both ends, the SSPPs’ transmission line can also filter out low frequencies. Step impedance branches and circular slits [20] at both ends of the SSPPs can be added to the transmission line. However, their special feeding method makes it have the effect of common mode rejection. This design method suppresses low frequencies through resonant units at both ends of the transmission line. The lower cutoff frequency is directly related to the size of the bandpass filter. This design method can also adjust the upper and lower cut-off frequencies separately. Refs. [21,22] proposed a method to directly design SSPPs as a T-shaped resonator to achieve a narrow bandpass and to achieve a tunable passband by introducing a varactor diode. However, this design method will lead to a large insertion loss, and the roll-off factor is not ideal at low frequencies.

At present, there is some research on the application of CSRRs as slow-wave periodic structures in recent years. Ref. [23] focuses on the discussion of the CSRR structure. The bandwidth of this structure needs to be improved. Ref. [24] adds an extra open-loop defected ground structure (DGS) to the serpentine design, which improves the standing wave performance in the low-frequency band. However, this structure cannot be applied in on-chip components since it requires etching of the ground plane.

With the development of the semiconductor integrated circuit industry, radio frequency devices are entering the era of miniaturized and integrated system-on-chip (SoC). The SiGe(Bi)-CMOS semiconductor processing technology provides a solution for the miniaturization of the filter. Compared with traditional microstrip and substrate integrated waveguide (SIW) millimeter-wave filters, the on-chip millimeter-wave filters have a higher degree of miniaturization and are much easier to integrate with other RF devices [25,26,27]. To realize notch-bands, Refs. [28,29,30] proposed the additional resonate structures using PCB technology. However, most of them can be applied under X-band due to manufacturing limits of PCBs.

Based on some current research, this paper proposes an SSPP filter loaded with CSRRs and serpentine slow-wave lines. First, we have made modifications to the original SSPP structure so that the bandwidth has been extended due to the increased slow-wave effect. Then, we have made topological changes to the CSRRs to reduce the cross-coupling. Therefore, from the simulated results, the notch-band can be tuned independently without much effect on the performances of the passband. The designed structure is based on the SiGe(Bi)-CMOS semiconductor processing technology with the operating frequency around 60 GHz. The proposed filter is designed, simulated, manufactured, and measured. A wide notch-band has been created and the measured results are in good agreement with the simulated ones.

## 2. Theory and Analysis

**Figure 1:**
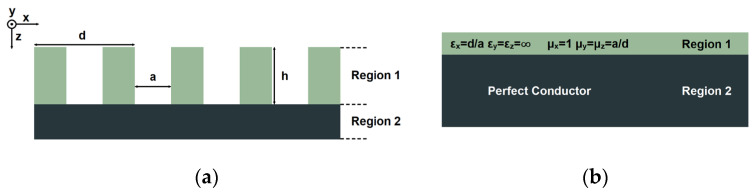
The SSPP structure: (**a**) physical structure and (**b**) equivalent artificial electromagnetic medium structure.

When exposed to microwave and millimeter-wave frequencies, metal surfaces can be considered excellent conductors. In order to have similar properties to surface plasmons, some holes and grooves can be loaded to the metal. From the perspective of artificial electromagnetic mediums, when the size of the grooves or holes is much smaller than the wavelength, through proper ranging of the grooves and holes, it can be regarded as a plasmonic material in the microwave or millimeter-wave region. A typical one-dimensional SSPP structure is shown in Figure 1, which consists of several metal grooves in region 1 and a metal stripe in region 2.

For the metal surface of the one-dimensional periodic grooves, the structure can be regarded as a new artificial electromagnetic medium layer with a uniform and anisotropic thickness covering the metal. The dielectric constant of this equivalent artificial electromagnetic medium can be given as [31]

(1) $$\varepsilon_{x}=\frac{d}{a}\;\varepsilon_{y}=\varepsilon_{z}=\infty$$

The propagation speed of the electromagnetic wave in the groove along the *y* and *z* directions is equal to the speed of electromagnetic waves in free space, so

(2) $$\sqrt{\varepsilon_{x}\mu_{y}}=\sqrt{\varepsilon_{x}\mu_{z}}=1$$

Therefore, the relative permeability in all directions can be obtained as

(3) $$\mu_{x}=1\;\mu_{y}=\mu_{z}=\frac{1}{\varepsilon_{x}}$$

Then, the reflection coefficient of a TM mode electromagnetic wave can be calculated as

(4) $$R=\frac{\left(\varepsilon_{x}k_{z}-k_{0}\right)+\left(\varepsilon_{x}k_{z}+k_{0}\right)e^{j2k_{0}h}}{\left(\varepsilon_{x}k_{z}+k_{0}\right)-\left(\varepsilon_{x}k_{z}-k_{0}\right)e^{j2k_{0}h}}$$

Since surface waves are bound to the surface of the structure and the condition *k_x_* > *k*_0_ stands, *k_z_* can be expressed as

(5) $$k_{z}=j\sqrt{k_{x}^{2}-k_{0}^{2}}$$

When the denominator of the expression of *R* reaches 0, the condition of the surface wave can be satisfied. At this time, the condition can be given as

(6) $$\frac{\sqrt{k_{x}^{2}-k_{0}^{2}}}{k_{0}}=\frac{a}{d}\tan\left(k_{0}h\right)$$

**Figure 2:**
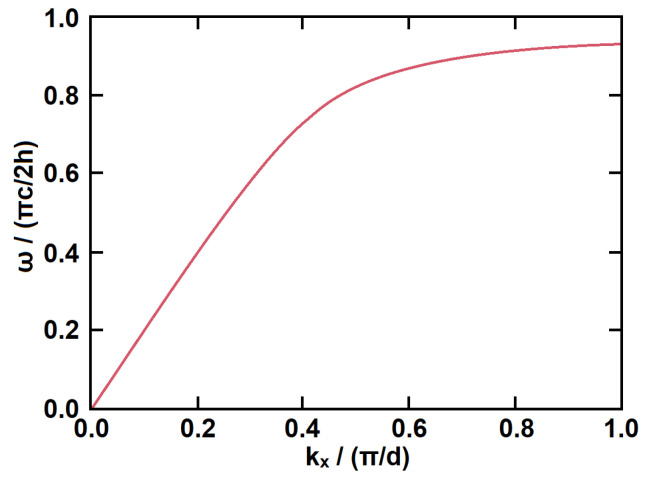
Dispersion curves of surface waves on one-dimensional metal structure with periodic grooves.

Figure 2 shows the dispersion relation between *k_x_* and *ω*, where *k_x_* is normalized to *π*/*d* and *ω* is normalized to *πc*/2*h*. The dispersion curve of this surface wave is very similar to that of surface plasmons at the metal/dielectric interface, confirming that SSPP structures can be regarded as surface plasmons in the microwave and millimeter-wave frequency region. In addition, SSPP structures have cut-off frequencies, and their dispersion is largely determined by the depth of the grooves.

**Figure 3:**
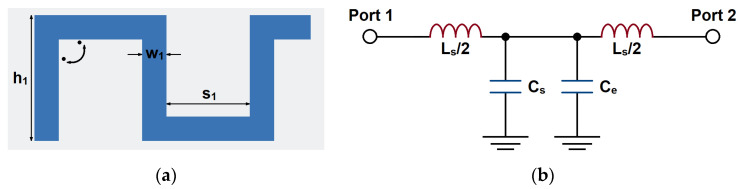
The serpentine structure: (**a**) topology and (**b**) equivalent circuit.

Transmission lines loaded periodically with reactive elements are periodic structures, which can be made in various forms related to the properties of the transmission line. The periodic serpentine lines can be used to increase the unit capacitance of the transmission line and increase slow-wave effects. The schematic diagram of the serpentine structure is shown in Figure 3. It is composed of multiple sub-branches with the gap *s*_1_, length *h*_1_, and line width *w*_1_. When the straight line is folded to the serpentine line, the extra electric coupling can be generated in the corner of the serpentine line. This can be regarded as a short segment of the self-coupling line; therefore, the distributed capacitance has been increased.

Since there is no major line width variation, it is equivalent to series inductance *L_s_* and capacitance to ground *C_s_*, as a T network. The corner can generate extra electric coupling, which can be regarded as an extra capacitor *C_e_*. The characteristic impedance of the line is *Z_s_* and the electrical length is *θ_s_*. According to the cascading principle, its ABCD matrix can be calculated as [32]

(7) $$\left[\begin{array}{cc} A & B\\ C & D \end{array}\right]=\left[\begin{array}{cc} 1 & j\omega \frac{L_{s}}{2}\\ 0 & 1 \end{array}\right]\left[\begin{array}{cc} 1 & 0\\ j\omega \left(C_{s}+C_{e}\right) & 1 \end{array}\right]\left[\begin{array}{cc} 1 & j\omega \frac{L_{s}}{2}\\ 0 & 1 \end{array}\right]=\left[\begin{array}{cc} 1-\omega \frac{L_{s}\left(C_{s}+C_{e}\right)}{2} & j\omega \frac{L_{s}}{2}\\ j\omega \left(C_{s}+C_{m}\right) & 1-\omega \frac{L_{s}\left(C_{s}+C_{e}\right)}{2} \end{array}\right]$$

Under the condition that the line is homogeneous, according to the analysis of the periodic structure by Floquet theory, the electrical length *θ_s_* = *βl*, where *β* is the phase constant of the line; the characteristic impedance *Z_s_* of the element in the passband can be expressed as

(8) $$\sin\beta l=A=1-\omega \frac{L_{s}\left(C_{s}+C_{e}\right)}{2}$$

(9) $$Z_{s}=\frac{B}{\sqrt{A^{2}-1}}=\sqrt{\frac{L_{s}}{\left(C_{s}+C_{e}\right)}}\cdot \sqrt{1-\frac{\omega^{2}L_{s}\left(C_{s}+C_{e}\right)}{4}}$$

Therefore, based on (8), the extra capacitance *C_e_* can be regarded as the increase in capacitance, which results in the decrease in *β*. Then, the slow-wave effect becomes stronger.

**Figure 4:**
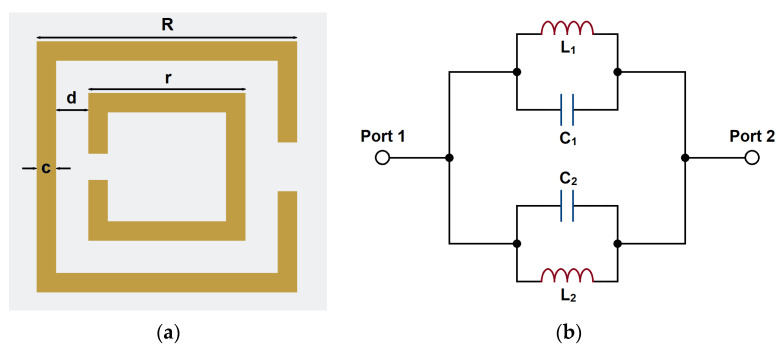
The structure of CSRR: (**a**) topology and (**b**) equivalent circuit model.

When the electromagnetic wave is propagating through the CSRR, the change of the electric field will cause an inductive effect on the internal and external metals of the structure. The groove between the internal and external metals will produce a capacitive coupling effect, thus forming an obvious electrical resonance effect. So, CSRR can be modeled with an equivalent inductor and capacitor. The equivalent circuit model of the CSRR structure is shown in Figure 4.

We have used the electromagnetic simulation software for full wave simulation. The software employs the finite element method to generate an electromagnetic field solution. In the finite element method, the full problem space is generally divided into thousands of smaller regions and represents the field in each sub-region with a local function. The geometric model is divided into a large number of tetrahedra, and the finite element mesh consists of this collection of tetrahedra, where a single tetrahedron is a four-sided pyramid. The finite element process to calculate the *S*-matrix associated with a structure with ports can be concluded as follows. Firstly, the structure is divided into finite element meshes. Then, it calculates the modes on each port of the structure supported by a transmission line with the same cross-section as the port. The next step is to calculate the full electromagnetic field pattern inside the structure and the generalized *S*-matrix from the amount of reflection and transmission that occurs. Finally, the magnitude of transmitted and reflected signals is calculated directly from a given set of input signals by the resulting *S*-matrix, which reduces the full 3D electromagnetic behavior of a structure to a set of high-frequency circuit parameters.

**Figure 5:**
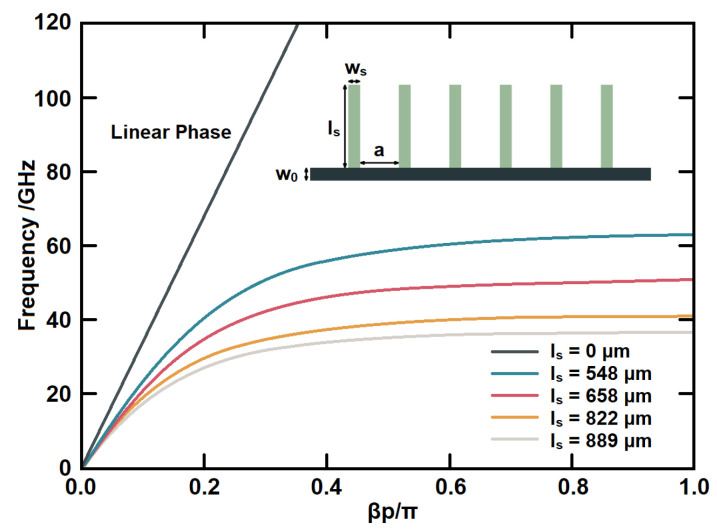
The dispersion curve of SSPP unit structure.

The SSPP units used in this design and their dispersion characteristics are shown in Figure 5. The dispersion effect increases with the length of *l_s_*, which results in a greater dispersion curve. Therefore, the electromagnetic waves can pass the structure within a certain resonant frequency for a coupling or resonant cavity. Therefore, the passband bandwidth can be compressed by utilizing the dispersion properties of SSPPs. Other dimensions are given as (in μm): *w*_0_ = 72, *a* = 300, and *w_s_* = 87.

**Figure 6:**
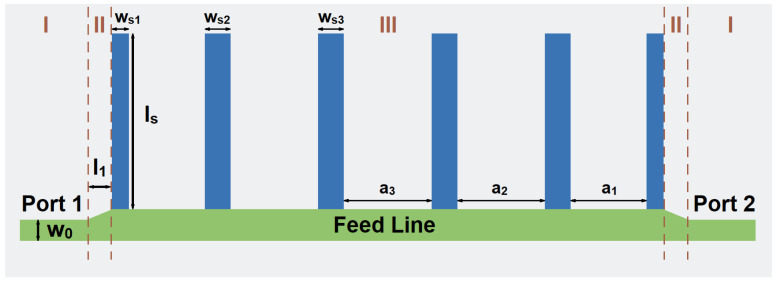
The topology of the high-order SSPP structure.

The designed structure based on SSPP is shown in Figure 6. It can be divided into three sections. The first part is the input and output lines with the characteristic impedance of 50 Ohm. The second part is the tapered lines to achieve impedance transition. This pair of structures ensures that the impedance from the input transitions smoothly to the SSPP and then to the output. The third part consists of five SSPP U-shaped unit cells. The topology is the upper part of the filter. The back plane is shown as the gray region and it is used as the ground.

In our simulation, we have set the solution type to modal. Then, the filter structure is plotted using the 0.13 μm SiGe (Bi)-CMOS substrate setup. The geometric values in each segment are set as variables for optimization. In order to feed the electromagnetic wave in the structure, the input and output ports are set as two wave ports. The next step is to set the driven solutions. We have used the adaptive solutions in broadband from 35 GHz to 75 GHz. The maximum number of passes for one set of geometric values is 30 with the maximum convergence threshold delta *S* of 0.01. During frequency sweep, we have used interpolating sweep type with 401 frequency points. Then, the simulation can be started. The field distributions and *S*-parameters can be obtained.

**Figure 7:**
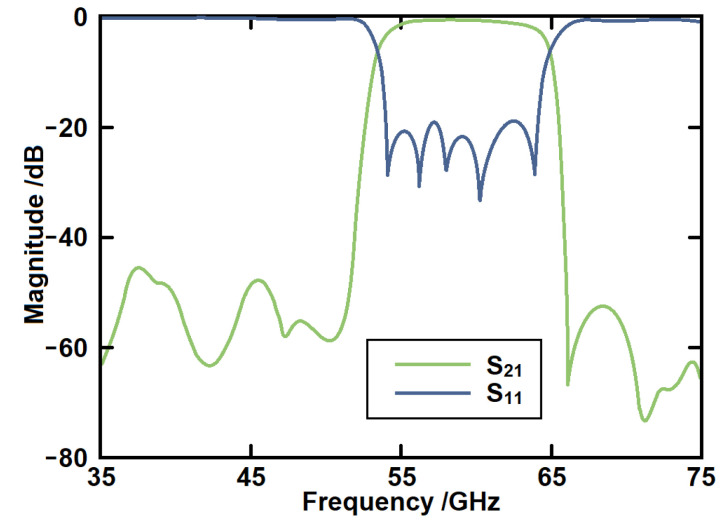
The simulated *S*-parameters of the high-order SSPP structure.

**Figure 8:**
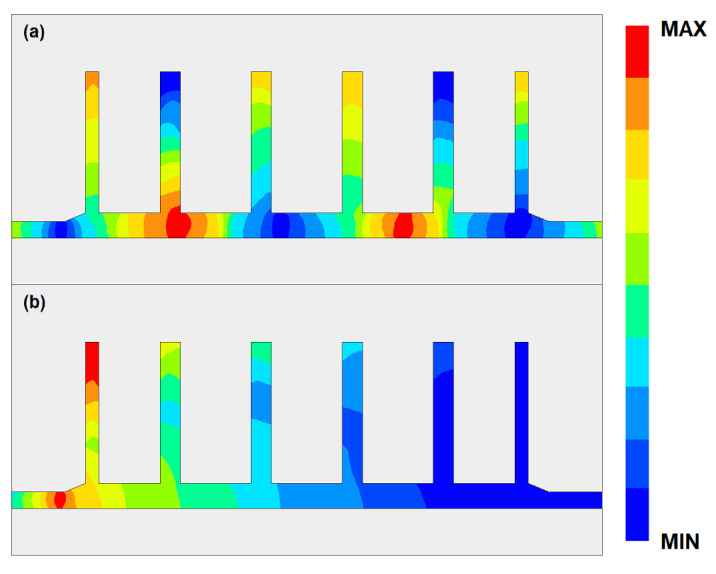
The field distribution of the SSPP structure at (**a**) passband and (**b**) stopband.

The simulated *S*-parameters are given in Figure 7. It can be seen that a passband from 53.4 GHz to 64.9 GHz has been generated. This passband is generated by higher order mode of the SSPP structure. The return loss in the band is better than 20 dB. The out-of-band rejection is over 40 dB. The in-band minimum insertion loss is about 0.6 dB. This structure has laid the foundation for our subsequently proposed structure. The field distributions of the SSPP structures in both passband and stopband are shown in Figure 8.

The filtering properties are produced by the tabs of the SSPP structure. At the passband, the condition of resonance is not satisfied; therefore, all the tabs can be regarded as open-ended lines. Then, the electromagnetic energy can pass through the feed line without much attenuation. However, at the stopband, the tabs start to resonate, and, based on the quarter-wave impedance transformation theory, the tabs now become short-ended lines. In this condition, the electromagnetic waves can be regarded as seeing a barrier and are reflected back to the feeding port. The tabs of SSPP act as switches with different states at different frequencies. From stopband to passband, the first cut-off frequency can be calculated as

(10) $$f_{c1}=\frac{1}{\pi \sqrt{L_{s}\left(C_{s}+C_{m}\right)}}$$

When switching to stopband again, the resonant mode moves from the basic mode to the first higher mode; therefore, the second cut-off frequency is mainly determined by the intrinsic reactance and can be calculated as

(11) $$f_{c2}=\frac{1}{2\pi \sqrt{L_{s}C_{s}}}$$

**Figure 9:**
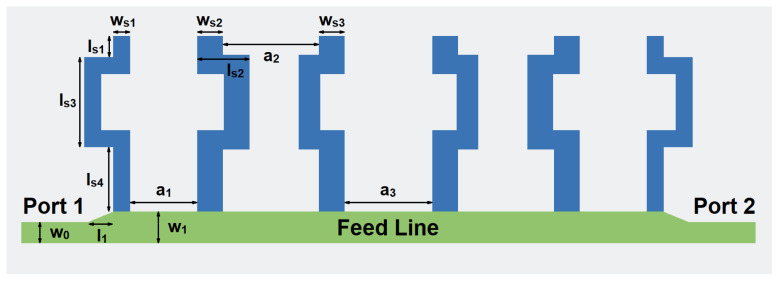
The topology of the SSPP structure with serpentine lines.

Based on our previous analysis, in order to increase the bandwidth, the lower transition band should be moved to the left region or the higher transition band should be moved to the right region. Therefore, we have modified the straight lines in the traditional SSPPs into the serpentine lines to increase the distributed capacitance and inductance along the lines, as shown in Figure 9. Therefore, the bandwidth can be increased by moving the lower transition band to the left region.

**Figure 10:**
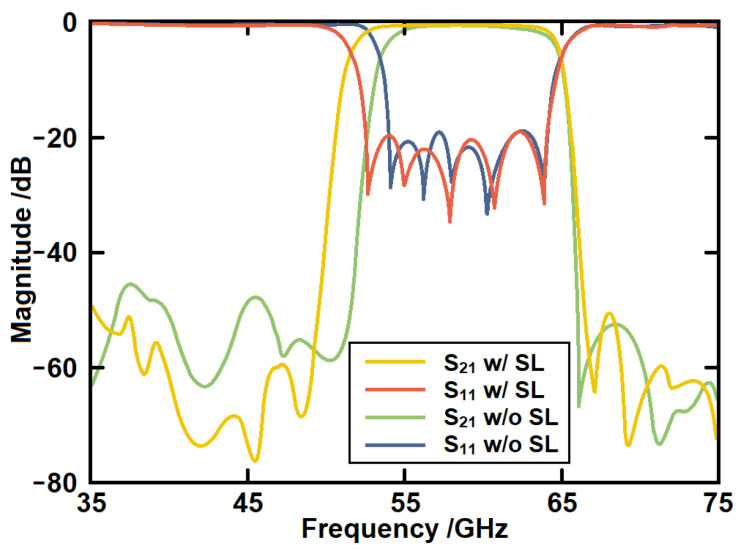
The simulated *S*-parameters of the SSPP structure with serpentine lines in comparison with the straight-line structure.

**Figure 11:**
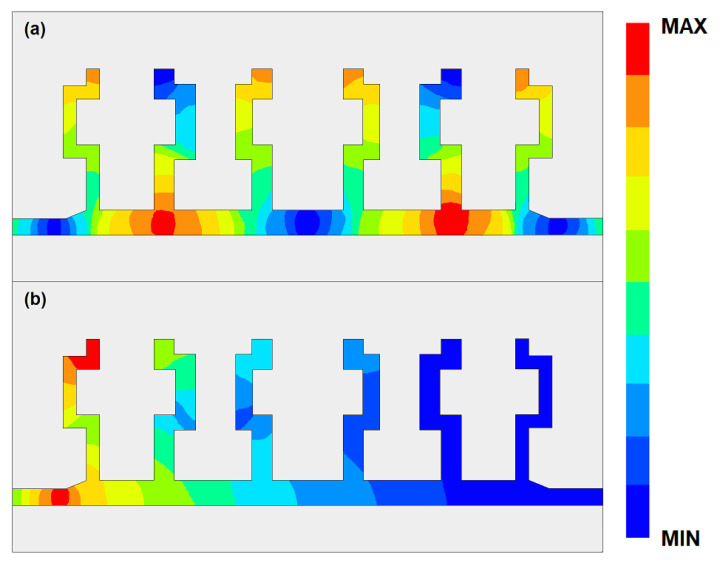
The field distribution of the modified SSPP structure with serpentine lines at (**a**) passband and (**b**) stopband.

The simulated results of the serpentine structure in comparison with the traditional structure are given in Figure 10. It can be seen that the lower transition band has been moved from 53.4 GHz to 51.5 GHz with little effect on the upper transition band. Therefore, the band width has been increased from 11.5 GHz to 13.4 GHz. The serpentine lines have another advantage. By changing the shapes of the lines, some gaps have been increased, and they can be used to place several CSRRs to form the flexible notch-band. The field distributions of the modified SSPP structure with serpentine lines in both passband and stopband are shown in Figure 11.

**Figure 12:**
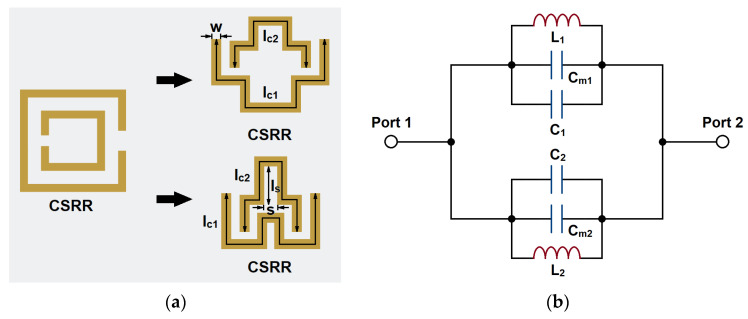
The shape modifications of the CSRR structure: (**a**) topology and (**b**) equivalent circuit model.

To better fit the CSRR structure into the gaps, we have made some shape modifications, as shown in Figure 12a. The CSRR structure consists of two open-ended lines. Each line can resonate alone to produce an independent transmission zero in the passband. Through the coupling of the two lines, the transmission zeros can produce a notch-band. This band can suppress the unwanted signals in the passband. Furthermore, the modifications have suppressed the parasitic effects by reducing the cross-coupling between the segments. The equivalent circuit model of the modified CSRR structure is shown in Figure 12b. In addition to the original equivalent capacitances and inductances, capacitances *C_m_*_1_ and *C_m_*_2_ have been created by the center convex shape. It can be regarded as a segment of parallel coupling lines shorted at one end. The electric coupling effect increases the equivalent capacitance, and therefore it can be regarded as additive capacitances. The increase in total capacitances can be seen as the reduction in CSRR size, since the equivalent capacitance is mostly proportional to the resonator size.

**Figure 13:**
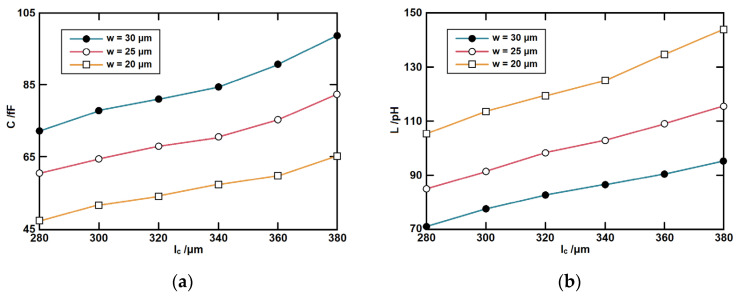

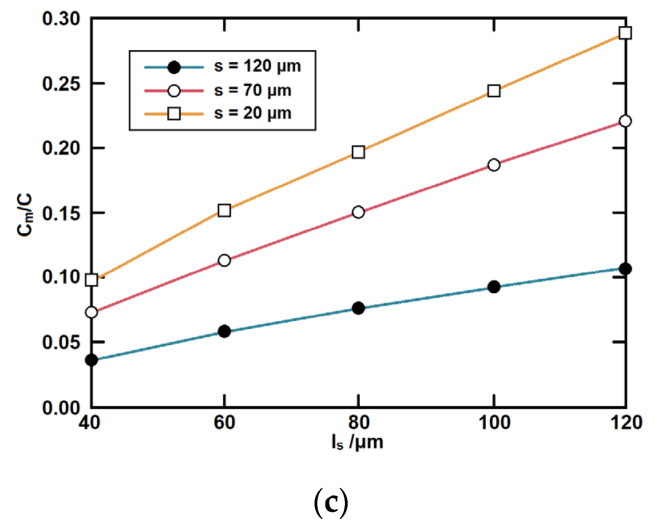
The component values with respect to dimensions (**a**) *C*, (**b**) *L*, and (**c**) *C_m_*.

The values of the equivalent *L* and *C* with the change of CSRR dimensions are given in Figure 13. These values are extracted based on full-wave simulations. *l_c_* and *w* are the length and width of the resonator. *l_s_* and *s* are the length and slot width of the coupling area. It can be seen that *C* increases with the increase in the length and width. *L* increases with the length but decreases with the width. *C_m_* increases with the coupling length but decreases with the coupling width.

**Figure 14:**
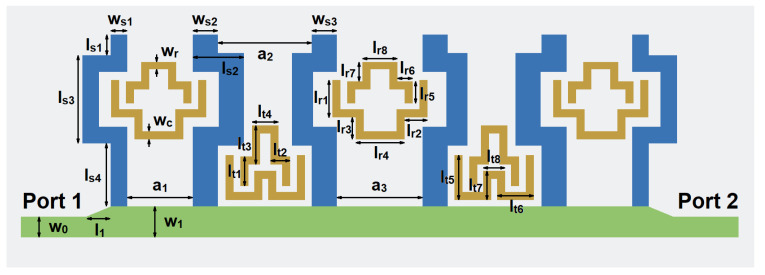
The proposed SSPP filter loaded with CSRRs.

Figure 14 shows our final proposed SSPP filter loaded with CSRRs. Between each wide gap, a CSRR structure is placed inside. Compared with the structure in [10], extra notch-band modes can be generated. Then, the bandwidth of the notch-band can be increased and also can be tuned independently during the design process. Thus, the design process can be facilitated.

During the design process, we have already performed the optimization process in consideration of both filter topology and performances. The minimum line width and slit width have been set according to the manufacturing requirement. All the dimensions have been set as variables. The maximum number of iterations is set at 100,000. The cost function is based on the requirements of *S*_21_ and *S*_11_. Furthermore, we have used different optimizers, such as the quasi-Newton method, adaptive multiple-objective method, and so on. The results given in the paper are the optimum values based on our simulations.

**Figure 15:**
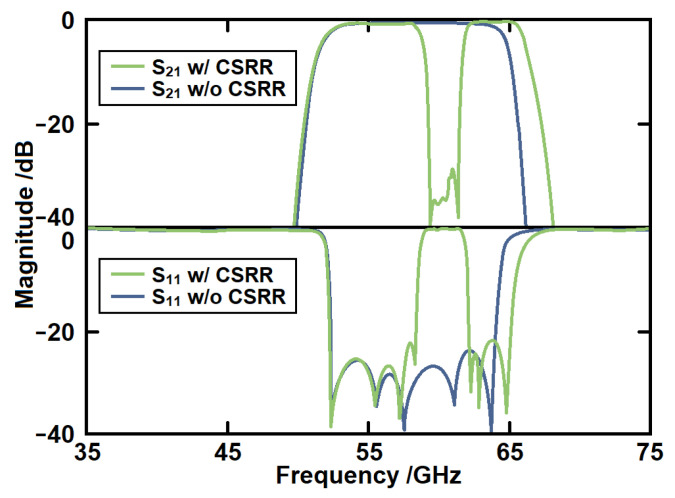
The simulated *S*-parameters of the serpentine line SSPP with and without CSRR structures.

Figure 15 gives the simulated *S*-parameters of the serpentine line SSPP with and without CSRR structures. First, it can be seen that a notch-band from 58.8 GHz to 61.7 GHz has been produced within the passband. The rejection level of the notch-band is over 30 dB. The second advantage is that the CSRR can form a weak coupling between the serpentine lines. Therefore, the upper transition band has been moved to a higher region. Then, the bandwidth has been further increased at the same time.

**Figure 16:**
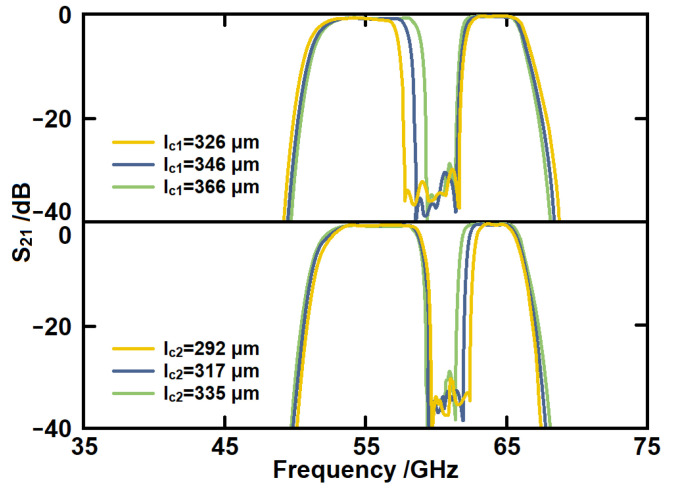
The simulated *S*_21_ of the proposed filter with different sizes of CSRRs.

Figure 16 gives the simulated *S*_21_ of the proposed structure with different lengths of the CSRR lines. The line *l_c_*_1_ is mainly responsible for the frequency of the lower notch frequency. With the increase in the *l_c_*_1_, the first notch-band frequency decreases without any influence on the second notch-band frequency. The line *l_c_*_2_ is mainly responsible for the frequency of the higher notch frequency. It can be observed that with the increase in *l_c_*_2_, the frequency of the second notch frequency decreases without any influence on the first notch frequency. Therefore, the bandwidth can be tuned independently, and this property can simplify the design process.

**Figure 17:**
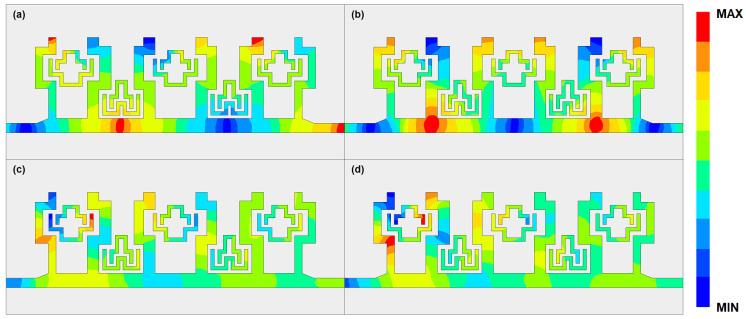
Schematic diagram of the field distribution of the proposed SSPP-SL-CSRR structure at (**a**) 54.3 GHz, (**b**) 63.2 GHz, (**c**) 59.0 GHz, and (**d**) 61.5 GHz.

Figure 17 gives the electric field distribution of the proposed filter at frequencies of 54.3 GHz, 63.2 GHz, 59.0 GHz, and 61.5 GHz. Since the CSRRs are added in the SSPP structures, a transmission stop band has been produced. The electric field tensity is over ten times weaker than that at the passband. The passband is mainly achieved through the transmission mode of the SSPP structure. Therefore, the insertion loss is relatively small in the passband. In the notch-band, the two segments of the CSRRs resonate, which reflects the electromagnetic wave energy back to port 1. This can be regarded as a parallel resonate LC circuit, where the input impedance becomes infinitely large from port 1 to port 2 at the resonate frequency. Therefore, it prohibits the transmission of the guided wave.

## 3. Manufacturing and Measurement

Most millimeter-wave filters are manufactured using printed circuit board (PCB) technology, low-temperature cofired ceramics (LTCC) technology, and substrate integrated waveguide (SIW) technology. Although conventionally fabricated millimeter-wave bandpass filters have good performance with low insertion loss and high frequency selectivity, the miniaturization of millimeter-wave filters is also limited due to the influence of the narrowest metal lines in these processes. With the development of semiconductor lithography technology, the manufacturing precision has reached the level of the micrometer and nanometer, which has provided the basis for further miniaturization of millimeter-wave bandpass filters. On the other hand, in pursuit of low power consumption and miniaturization of systems, more and more devices are integrated into chips to build system-on-chips (SoCs). Therefore, the advantages of on-chip millimeter-wave bandpass filters have become increasingly prominent.

**Figure 18:**
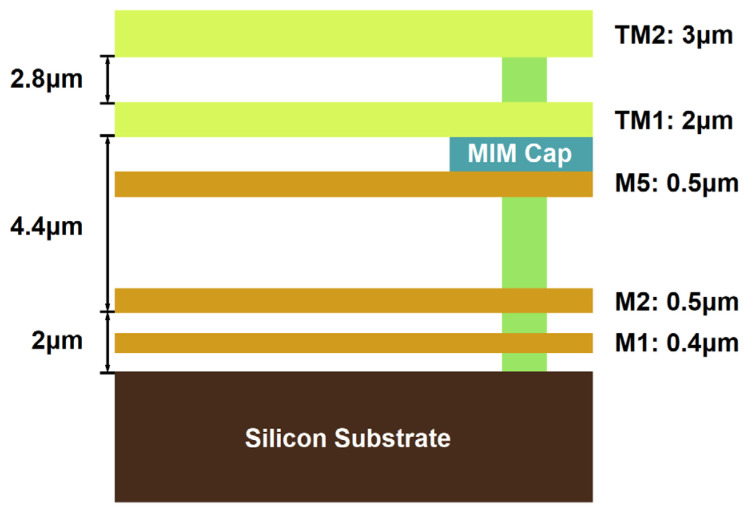
The structural diagram of 0.13 μm on-chip SiGe process.

In order to meet the requirement of the proposed design, we have adopted the 0.13 μm SiGe processing technology to design the filter. A schematic diagram of the multi-layer stack of 0.13 μm SiGe is given in Figure 18. This manufacturing process consists of seven layers of metal. The TM2 layer and the TM1 layer are comparatively thicker metal layers, which have relatively small metal loss compared with other metal layers. In addition, six through-hole layers can be used for interlayer connection. According to semiconductor processing rules, metal layers and metal vias connected to the layers are designed in a silicon dioxide layer, and the silicon substrate is below the silicon dioxide layer. The relative permittivity of silicon dioxide is 4.1, with a loss tangent of 0.01. The height of the silicon substrate is 200 μm. The dimensions of the filters are given as (in μm): *w_r_* = *w_c_* = 25, *w_s_*_1_ = 58, *w_s_*_2_ = *w_s_*_3_ = 87, *w*_0_ = 72, *w*_1_ = 107, *l*_1_ = 81, *l_s_*_1_ = 72, *l_s_*_2_ = 177, *l_s_*_3_ = 305, *l_s_*_4_ = 218, *a*_1_ = 227, *a*_2_ = 325, *a*_3_ = 297, *l_r_*_1_ = 129, *l_r_*_2_ = 79, *l_r_*_3_ = 76, *l_r_*_4_ = 170, *l_r_*_5_ = 76, *l_r_*_6_ = 53, *l_r_*_7_ = 67, *l_r_*_8_ = 121, *l_t_*_1_ = 103, *l_t_*_2_ = 68, *l_t_*_3_ = 135, *l_t_*_4_ = 92, *l_t_*_5_ = 156, *l_t_*_6_ = 128, *l_t_*_7_ = 101, and *l_t_*_8_ = 76.

**Figure 19:**
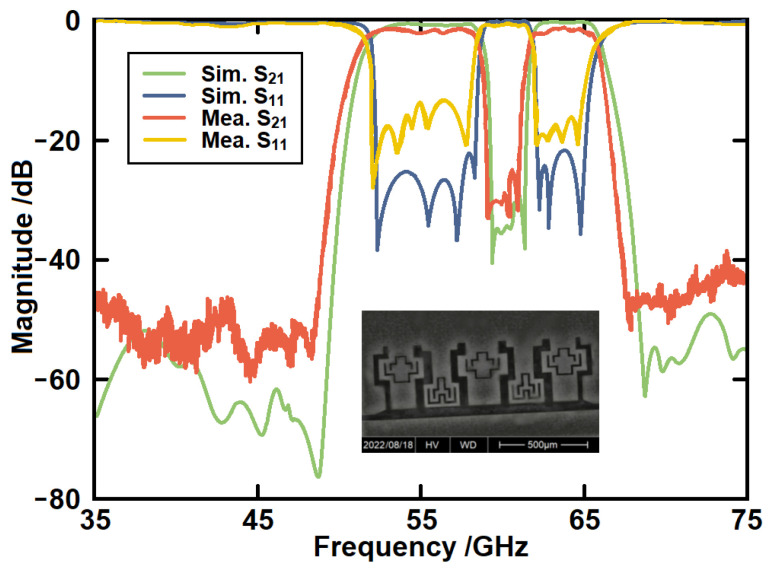
The simulated and measured results of the proposed filter.

The measured results in comparison with the simulated ones are given in Figure 19. The proposed on-chip filter is measured through a pair of G-S-G probes connected with a vector network analyzer. The die photo is shown in the inset. It can be observed that a passband from 51.5 GHz to 65.8 GHz has been produced. The measured in-band insertion loss is 2.6 dB. A notch-band is from 58.4 GHz to 61.7 GHz with the rejection level over 25 dB. The measured out-of-band rejection is higher than 40 dB. The insertion loss increases with the rise in frequency. The measured results are generally in good agreement with the simulated results. Table 1 gives the performance of some recently published on-chip filters. It can be seen that comparable performances have been achieved.

Between the simulated and measured results, the passband frequency, bandwidth, and notch-band are in good agreement. There is no major frequency shift between the simulation and measurement. The discrepancies exist in several parts: in-band insertion loss, in-band return loss, and out-of-band suppression. We have simulated the filter using the HFSS material library where the materials are treated as ideal materials. During manufacturing, the situation cannot be that ideal. Therefore, the loss has been increased in the real situation. This decreases the quality factors of the resonators, which worsens in-band return loss and out-of-band suppression at the same time. Manufacturing precision and packaging effects can also increase the discrepancy. The simulation is not able to take all the loss in the real situation into account. The measured results can be more trusted.

**Table 1:** Comparison of some recently published filters.

Ref.	Passband Range (GHz)	Passband Bandwidth (GHz)	Notch-Band Bandwidth (GHz)	In-Band Insertion Loss (dB)	Out-of-Band Rejection (dB)	Notch-Band Suppression (dB)	Size (λ_g_ × λ_g_)	Number of Out-of-Band TZs	Manufacturing Technology
[25]	18.1~20.3	2.2	/	0.9	37	/	0.26 × 0.21	2	0.13 μm Bulk CMOS Technology
[26]	20.75~41	20.25	/	3.6	>35	/	0.28 × 0.22	2	0.13 μm BiCMOS Technology
[27]	55.6~79.4	23.8	/	1.7	>40	/	0.92 × 0.26	2	0.15 μm GaAs PHEMT technology
[28]	2.2~4.1	1.9	0.2	1.6	>15	40	0.62 × 0.48	5	High-speed PCB Technology
[29]	0.87~1.4	0.53	<0.1	1	>15	39~55	0.61 × 0.44	7	High-speed PCB Technology
[30]	1.2~2.36	1.16	0.09~0.2	0.8	>30	2~79	0.9 × 0.45	9	High-speed PCB Technology
Proposed	51.5~65.8	14.3	3.3	2.6	>40	>30	0.71 × 0.22	6	0.13 μm on-chip SiGe Technology

## 4. Conclusions

This paper proposed an SSPP filter with CSRRs based on the 0.13 μm on-chip SiGe technology. The filter has realized the passband in the unlicensed frequency region of 5G communication systems. A wide flexible notch-band has also been produced within the passband to suppress in-band interferences. The starting and ending frequencies can be independently designed by tuning the segments of the CSRR structures. The proposed filter has been simulated, manufactured, and measured, and the measurement results are in good agreement with the simulated ones. Compared with some on-chip filters in other literature, the proposed filter shows excellent performance.

## Data Availability

Data available on request due to privacy. The data presented in this study are available on request from the corresponding author.
